# Overexpression of lncRNA SLC16A1-AS1 Suppresses the Growth and Metastasis of Breast Cancer *via* the miR-552-5p/WIF1 Signaling Pathway

**DOI:** 10.3389/fonc.2022.712475

**Published:** 2022-03-15

**Authors:** Bin Jiang, Jie Xia, Xudong Zhou

**Affiliations:** ^1^ Department of Thyroid and Breast Surgery, Tongji Hospital, Tongji Medical College of Huazhong University of Science and Technology, Wuhan, China; ^2^ Department of Respiratory and Critical Care Medicine, Tongji Hospital, Tongji Medical College, Huazhong University of Science and Technology, Wuhan, China; ^3^ Department of Surgery, Tongji Hospital, Tongji Medical College, Huazhong University of Science and Technology, Wuhan, China

**Keywords:** breast cancer, SLC16A1-AS1, miR-552-5p, WIF1, ceRNA

## Abstract

**Background:**

Breast cancer (BC) is the most common cancer and the fifth leading cause of cancer mortality with 685,000 deaths worldwide in 2020. Long non-coding RNAs (lncRNAs) are critical in BC carcinogenesis and progression. However, the functional roles and mechanisms of SLC16A1-AS1 in BC are unknown.

**Methods:**

The expression profile of SLC16A1-AS1 in BC patients was investigated using data from The Cancer Genome Atlas (TCGA) database and checked in 80 BC patients, followed by analyzing the prognostic value of SLC16A1-AS1 in the 80 BC patients. The biological functions of SLC16A1-AS1 were further examined *in vivo* and *in vitro* after overexpression of SLC16A1-AS1 in BC cells. Possible binding sites between SLC16A1-AS1 and miR-552-5p were predicted by miRDB and those between miR-552-5p and Wnt inhibitory factor-1 (WIF1) were predicted by miRanda, which were confirmed using dual-luciferase reporter assay with mutation. Spearman correlation assay was applied to evaluate the association between genes. Rescue experiments were further applied to investigate the molecular mechanisms involved.

**Results:**

Lower SLC16A1-AS1 expression in BC tissues was related to poor prognosis of BC patients. Upregulation of SLC16A1-AS1 suppressed BC cell viability, colony formation, invasion, and migration *in vitro* and growth *in vivo via* sponging miR-552-5p to release WIF1.

**Conclusion:**

SLC16A1-AS1 is a tumor suppressor in BC, and lower SLC16A1-AS1 expression is an indicator of poor prognosis in BC patients. SLC16A1-AS1 inhibits BC carcinogenesis and progression *via* the SLC16A1-AS1/miR-552-5p/WIF1 pathway. SLC16A1-AS1 represents a novel diagnostic, therapeutic, and prognostic target for BC management.

## Background

Breast cancer (BC) is the most common malignancy in women worldwide. It is predicted that there will be 2.3 million new cases of patients with BC, representing 11.7% of the total cancer patients. Meanwhile, BC accounts for one in four cancer cases, ranking first for incidence in 159 of 185 countries in 2020 (1). Despite the developments in BC diagnosis and treatments, the prognosis of BC patients is still unsatisfactory. BC is the fifth leading cause of cancer mortality with 685,000 deaths globally and accounts for one in six cancer deaths, ranking first for mortality in 110 of 185 countries in 2020 (1, 2). Consequently, a comprehensive understanding of the mechanisms involved in BC carcinogenesis and metastasis is crucial to improve the clinical outcomes of BC patients.

Recently, the application of high-throughput technologies in the life science field revealed that human genomes lacking protein-coding capacity could be transcribed into a variety of RNAs, such as long non-coding RNAs (lncRNAs) (2–4). Many lncRNAs function as regulatory factors in BC carcinogenesis and metastasis *via* diverse mechanisms (2, 4). For example, increased LINC01271 expression was related to metastasis and the unsatisfactory prognosis of BC patients (4). HOTAIR was upregulated and related to the development of BC patients, which regulates histone methylation to control gene expression in BC cells (5). However, the exact functions, clinical implications, and molecular mechanisms of the majority of lncRNAs in BC are basically unknown (2, 4).

Accumulating evidence suggested that lncRNAs in the cytoplasm mainly functioned as competing endogenous RNAs (ceRNAs), and they competed with microRNAs (miRNAs) to regulate the expressions of mRNAs (8). By competitively binding to their shared miRNA response elements (MREs), both mRNA and lncRNA expressions were regulated (6, 7). lncRNA-introduced ceRNA regulation has been involved in BC. For instance, BCRT1 was a ceRNA of miR-552-5p to promote BC development *via* targeting the miR-1303/PTBP3 axis (2).

**Figure 1:**
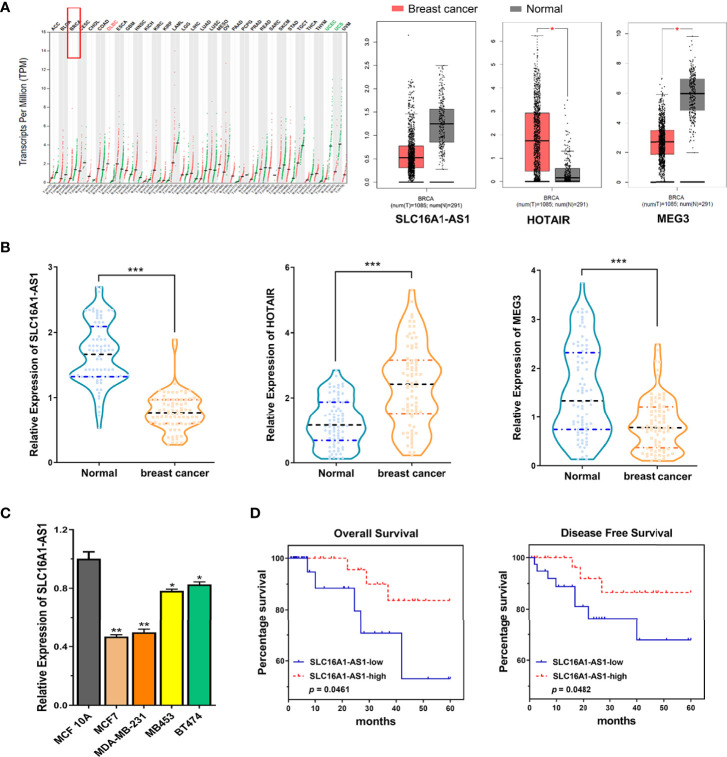
Expression of SLC16A1-AS1 is significantly downregulated in breast cancer (BC) cells and tissues and related to the prognosis of BC patients. **(A)** Bioinformatics analysis found downregulated SLC16A1-AS1 and MEG3 but upregulated HOTAIR expressions in tumor (T) tissues versus normal (N) tissues of BC (BRCA) patients by investigating the TCGA datasets using the online tool GEPIA. **(B)** SLC16A1-AS1 and MEG3 expressions were meaningfully downregulated, while HOTAIR expression was meaningfully upregulated in BC tissues versus paracancerous tissues from 80 BC patients analyzed by the qRT-PCR assay. **(C)** SLC16A1-AS1 expression was meaningfully lower in human BC (MCF7, MDA-MB-231, MB453, and BT474) than in human normal mammary gland epithelial cells (MCF 10A) analyzed with qRT-PCR, and the difference was most significant in MCF7 and MDA-MB-231 cells. **(D, E)** Analyzed by the Kaplan–Meier method among the 80 BC patients, lower SLC16A1-AS1 expression indicated a poor prognosis both in overall survival **(D)** and disease-free survival **(E)**. ^*^ *p* < 0.05, ^**^ *p* < 0.01, ^***^ *p* < 0.001.

As a biomarker, SLC16A1-AS1 has been reported in hepatocellular carcinoma (HCC), cervical squamous cell carcinoma (CSCC), glioblastoma, bladder cancer, oral squamous cell carcinoma (OSCC), and non-small cell lung cancer (NSCLC). However, SLC16A1-AS1 has been reported to promote (such as glioblastoma) or inhibit (such as CSCC) carcinogenesis in different cancers (8–12). Nevertheless, the function of SLC16A1-AS1 in BC remains unclear. By analyzing The Cancer Genome Atlas (TCGA) datasets with GEPIA online tool (http://gepia.cancer-pku.cn/index.html), SLC16A1-AS1 has been identified to be downregulated in BC (referred to as BRCA in GEPIA) tissues (**Figure 1A**). lncRNA HOTAIR (13) and MEG (14) are the recognized oncogene and tumor-suppressor gene in BC tissues (**Figure 1A**). The data reliability of LC16A1-AS1 expression has been further validated in BC tissues of selected patient samples using lncRNA HOTAIR and MEG as reference genes (**Figure 1B**). Therefore, we hypothesized that SLC16A1-AS1 might be a potential tumor suppressor in BC, and further investigated its regulatory function using BC cell and xenograft nude mice model. Our study might provide novel insights into BC progression involving the lncRNA and a potential new therapeutic target for malignancy.

## Patients and Methods

### Patient Specimens

Human BC and self-matched paracancerous tissues were collected from patients in our hospital during surgical treatment and then deposited in a refrigerator at −80°C after being frozen in liquid nitrogen.

### Reagents

The following were used in the study: Dulbecco’s modified Eagle’s medium (DMEM), lipofectamine 2000, pmirGLO plasmid, Fetal bovine serum (FBS), pcDNA3.1 plasmid and Trizol reagent (Invitrogen, Carlsbad, US); matrigel (BD, New Jersey, US); SurePrep™ Nuclear or Cytoplasmic RNA Purification Kit, PrimeScrip™ RT Master Mix, SYBR Premix Ex Taq I and PrimeScript miRNA cDNA Synthesis Kit (TaKaRa, Shiga, Japan); protease inhibitor cocktail (Sigma, St. Louis, US); Dual-Luciferase Reporter Assay System (Promega, Madison, US); CCK-8 test kit (Dojindo Corp, Kyushu, Japan); antibodies against WIF1(#5652), Ki-67(#9449) and β-Actin (#4967) (Cell Signaling Technology, Danvers, US).

### Informed Consent

Each included BC patient provided written informed consent. This research got permission from the Medical Ethics Committee of Tongji Hospital, Tongji Medical College following the Helsinki Declaration (TJ-IRB20180322).

### Cells and Cell Culture

A human normal mammary gland epithelial (MCF 10A) and four human BC (MCF7, MDA-MB-231, MB453, and BT474) cell lines were provided by the American Type Culture Collection (Manassas, USA) and maintained in DMEM containing 100 μg/ml streptomycin, 100 U/ml penicillin, and 10% FBS in a 37°C incubator with 5% CO_2_. For MCF 10A cells, horse serum (5%), EGF (20 ng/ml), hydrocortisone (0.5 μg/ml), cholera toxin (100 ng/ml), and insulin (10 μg/ml) were supplemented.

### Plasmids and Transfection

Full-length cDNA of SLC16A1-AS1 was cloned into pcDNA3.1 plasmid. Empty (NC) and SLC16A1-AS1 containing pcDNA3.1 (SLC16A1-AS1) plasmids were transfected into MCF7 and MDA-MB-231 cells, respectively, using Lipofectamine 2000 reagent. To screen stably transfected cells, 2 mg/ml of G418 was used. The pLKO.1 plasmid served as a negative control (NC) for WIF1 silencing. SLC16A1-AS1 or WIF1 3′-UTR sequences covering mutant (mut) or wild-type (WT) binding sites of miR-552-5p were separately cloned into dual-luciferase reporter plasmid (pmirGLO).

Three siRNAs and NC siRNAs for SLC16A1-AS1 were constructed by GenePharma (Shanghai, China) and transfected into MDA-MB-453 and BT474 cells, respectively. Lipofectamine 2000 was used to transfect NC, SLC16A1-AS1 siRNAs, or miR-552-5p mimics.

### Dual-Luciferase Reporter Analysis

Based on bioinformatics analysis, miR-552-5p could possibly bind to WIF1. pmirGLO Dual-Luciferase miR Target Expression Vector was applied to validate the direct binding between miR-552-5p and SLC16A1-AS1 3′-UTR (or WIF1 3′-UTR). Mut reporter constructs of pmirGLO/SLC16A1-AS1 3′-UTR-mut (or WIF1 3′-UTR-mut) and WT reporter constructs of pmirGLO/SLC16A1-AS13′-UTR (or WIF1 3′-UTR) were co-transfected together with miR-552-5p NC or miR-552-5p mimic in MCF7 and MDA-MB-231 cells. Firefly luciferase activity was determined using a microplate reader, and the final luciferase activity of the reported gene to be tested was calculated by normalizing to Renilla luciferase activity.

### Total RNA Extraction and qRT-PCR Assay

TRIzol reagent was applied for total RNA extraction. NanoDrop (ND-1000 model, Thermo Fisher Scientific, USA) was used to check RNA concentration and purity. The cDNA was reverse transcribed using the PrimeScript™ RT Master Mix. Reverse transcription was performed for miRNAs using the PrimeScript miRNA cDNA Synthesis Kit. Relative RNA level was calculated using the conventional 2^−ΔΔCt^ method. Real-time PCR primers for the target genes are shown in **Supplementary Table 1**.

### Nuclear and Cytoplasmic RNA Fraction Purification

The nuclear and cytoplasmic RNAs were separated with the SurePrep™ Nuclear or Cytoplasmic RNA Purification Kit following the manufacturer’s guideline. In brief, 80% confluent cells to be tested were lysed in a lysis solution, centrifuged for 3 min at 12,000 rpm to separate the cytoplasmic (supernatant fraction) and nuclear (pellet fraction) RNAs, followed by enrichment and elution on columns, respectively. The qRT-PCR assay was applied to determine SLC16A1-AS1 expression in the cytoplasm and nucleus using U6 and GAPDH as internal controls for nuclear and cytoplasmic RNAs, respectively.

### Cell Viability Assessment

Following the manufacturer’s guideline, 10 μl of CCK-8 solution was added into a 100-μl medium in cells cultured in a 96-well cell culture plate, and a viability curve was then built based on optional density (OD) values.

### Transwell Invasiveness and Migration Assays

The 24-well Transwell chambers (Corning Costar, USA) with or without Matrigel coating were applied to evaluate invasiveness or migration ability, respectively. Briefly, 750 μl of 20% FBS supplied medium was added into the lower chamber as a chemoattractant, and 200 μl of serum-free medium (3 × 10^5^ cells) was added into the upper insert and cultured for 48 h (for invasiveness assay) or 24 h (for migration assay). Cells on the lower surface of the upper insert were fixed with 4% paraformaldehyde and stained with 0.1% crystal violet.

### Colony Formation Evaluation

Cells to be tested were seeded (1,000/well) in a six-well plate and cultured for 7 days to form the cell colonies, followed by fixing for 10 min with 4% paraformaldehyde and staining for 5 min with 0.5% crystal violet. Colony images and numbers were acquired using a light microscope and ImageJ software.

### Western Blot Evaluation

NP40 lysis buffer with protease inhibitor cocktail was applied to lyse the cells. The same amounts of protein (20 μg) were isolated on SDS-PAGE gel, followed by transferring into a PVDF membrane, blocking with 5% non-fat milk, and incubating with primary and secondary antibodies.

### Animal Experiments

Twenty-four 6-week-old female BALB/c nude mice were bought from Beijing HFK Bioscience Co. Ltd. (Beijing, China), separated into four groups (*n* = 6) and maintained in the animal facility of our hospital. All *in-vivo* investigational procedures were approved and permitted by the Animal Care and Use Committee of our hospital. MCF7 and MDA-MB-231 cells overexpressing SLC16A1-AS1 or NC were suspended in PBS, respectively, and injected subcutaneously (1 × 10^7^ cells/mouse/200 μl) into the flank of each mouse. The minimum (*W*) and maximum (*L*) tumor lengths were checked with a vernier caliper every week to calculate the tumor volumes with the following formula: ½*LW* ^2^. The mice were sacrificed after 3 weeks. This study was approved by the Medical Ethics Committee of Tongji Hospital, Tongji Medical College (TJ-20200421).

### Immunohistochemical Assay

Paraffin-embedded sections were dewaxed with xylene, followed by rehydrating with alcohol, blocking endogenous peroxidase with 3% H_2_O_2_, antigen retrieval by microwave heating, blocking non-specific antigen in 5% BSA, incubating with a primary antibody against Ki-67 and then with a secondary antibody, staining using diaminobenzidine, and counterstaining using hematoxylin. Images were photographed under a microscope.

### Statistics Analysis

Student’s *t*-test or one-way ANOVA by SPSS 20.0 (IBM, Chicago, USA) was used for two- or multiple-group comparison, respectively. The Kaplan–Meier method with log-rank test was applied for survival rate calculation. Spearman correlation coefficient analysis was used to evaluate the association between genes. *p <*0.05 means statistical significance.

## Results

### Downregulated SLC16A1-AS1 Is Identified in Both BC Patient Tissues and Cells, and Lower SLC16A1-AS1 Expression Is Associated With Unsatisfactory Prognosis of BC Patients

To investigate the potential participation of SLC16A1-AS1 in BC carcinogenesis and development, we first investigated the lncRNA expression pattern based on public BC (BRCA) databases (TCGA dataset) by the online tool GEPIA, which showed a significantly lower SLC16A1-AS1 expression in tumor (T, *n* = 1,085) tissues than in normal (N, *n* = 291) tissues of BC (BRCA) patients (**Figure 1A**). Furthermore, to confirm the above findings, BC and self-matched paracancerous tissues and the clinicopathologic characteristics (**Supplementary Table 2**) of 80 BC patients were collected, which showed a significantly lower SLC16A1-AS1 expression in BC tissues than in paracancerous normal tissues (**Figure 1B**). The downregulated SLC16A1-AS1 expression was further confirmed in BC cells, which showed that SLC16A1-AS1 expression was considerably lower in MCF7, MDA-MB-231, MB453, and BT474 cells versus MCF 10A cells (**Figure 1C**), and SLC16A1-AS1 expression was the lowest in MCF7 and MDA-MB-231 cells. After the 80 BC patients were divided into SLC16A1-AS1 low-expression (*n* = 40) and SLC16A1-AS1 high-expression (*n* = 40) groups with the cutoff value of SLC16A1-AS1 median expression in BC tissues, the survival curves of the BC patients with SLC16A1-AS1 high-expression and SLC16A1-AS1 low-expression levels were plotted by Kaplan–Meier analysis, which showed a poor prognosis in BC patients with lower SLC16A1-AS1 expression (**Figure 1D**). The relationship between SLC16A1-AS1 expression level and the clinicopathologic characteristics of BC patients analyzed by the chi-square test showed that the SLC16A1-AS1 expression was statistically significantly related to tumor size, TNM stage, lymph node metastasis, and WIF1 expression but not related to age, gender, and tumor differentiation of patients (**Supplementary Table 2**).

### Overexpression of SLC16A1-AS1 Inhibits Viability, Colony Formation, Invasion, and Migration in BC Cells

**Figure 2:**
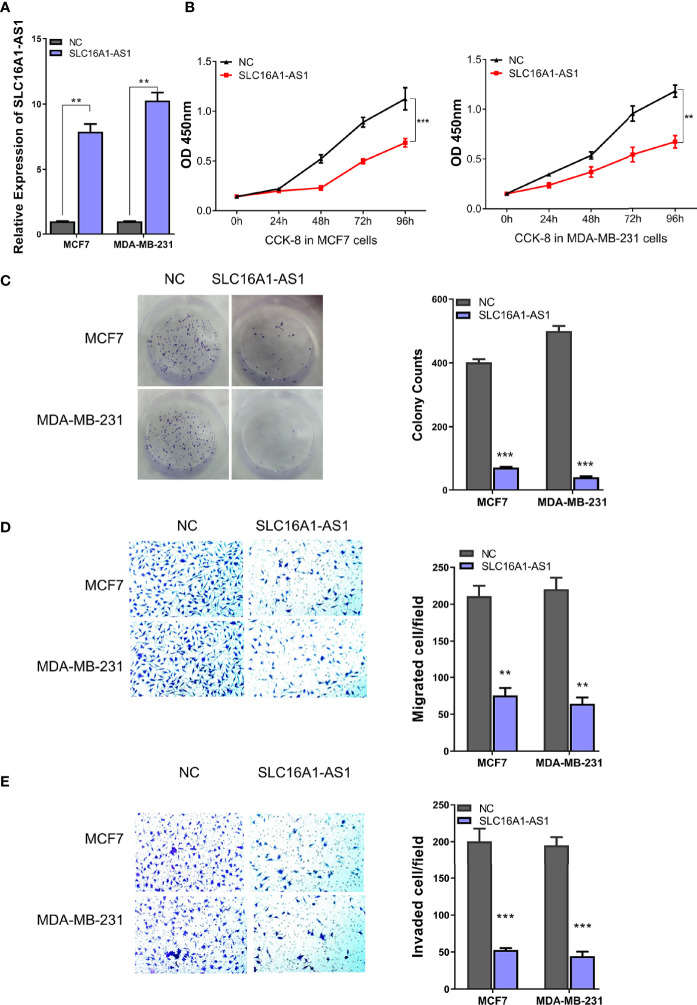
Silencing SLC16A1-AS1 promotes viability, colony formation, invasion, and migration of BC cells. To further explore the functions of SLC16A1-AS1 in BC progression, SLC16A1-AS1 was silenced in MDA-MB-453 and BT474 cells. After being confirmed that SLC16A1-AS1 was effectively silenced in both MDA-MB-453 and BT474 cells **(A)**, the following behaviors were compared between SLC16A1-AS1 silenced and the negative control (NC) MDA-MB-453 and BT474 cells: cell viability **(B)**, colony formation **(C)**, cell migration **(D)**, and cell invasiveness **(E)** in Figures 2 all were quantified with symbol *. ^*^ *p* < 0.05, ^**^ *p* < 0.01, ^***^ *p* < 0.001.

To determine the functions of SLC16A1-AS1 in BC cells, SLC16A1-AS1 was successfully overexpressed after transfection of pcDNA3.1 plasmid containing the SLC16A1-AS1 sequence in MCF7 and MDA-MB-231 cells, which was confirmed by qRT-PCR assay (**Figure 2A**). After SLC16A1-AS1 overexpression, the CCK-8 assay evaluating viability (0–96 h), the colony formation assay evaluating colony proliferation ability, and Transwell assays evaluating cell invasion and migration abilities were carried out. The data indicated that SLC16A1-AS1 overexpression significantly suppressed cell viability time dependently (**Figure 2B**) as well as colony proliferation ability (**Figure 2C**) and cell migration (**Figure 2D**) and invasion (**Figure 2E**) abilities in both MDA-MB-231 and MCF7 cells.

**Figure 3:**
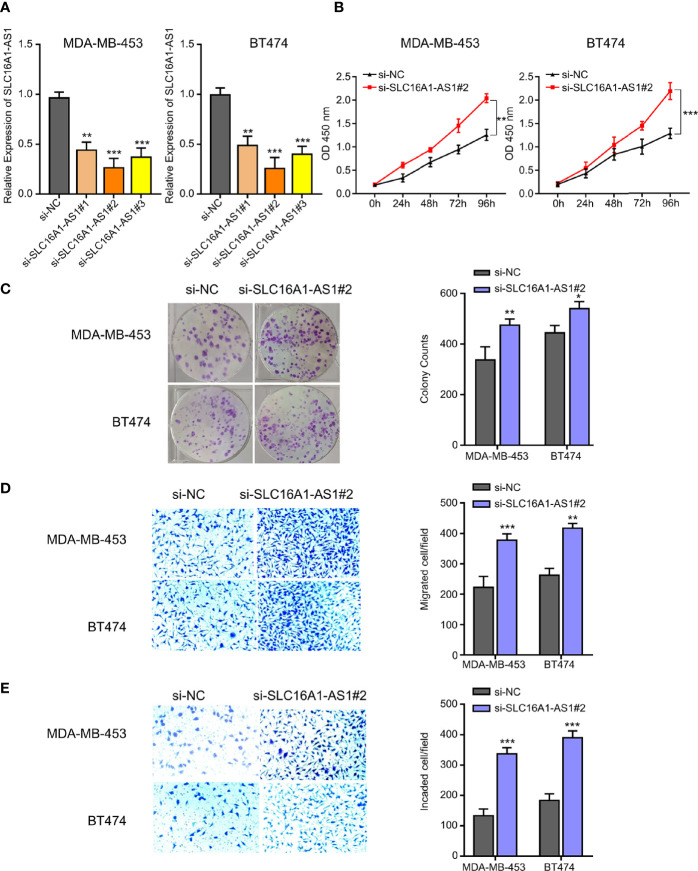
Overexpression of SLC16A1-AS1 inhibits the viability, colony formation, invasion, and migration of BC cells. To explore the functions of SLC16A1-AS1 in BC progression, SLC16A1-AS1 was overexpressed in MDA-MB-231 and MCF7 cells. After being confirmed that SLC16A1-AS1 was effectively overexpressed in both MDA-MB-231 and MCF7 cells **(A)** (*p *< 0.001), the following behaviors were compared between SLC16A1-AS1 overexpressed (SLC16A1-AS1) and the negative control (NC) MCF7 and MDA-MB-231 cells: cell viability **(B)**, colony formation **(C)**, cell migration **(D)**, and cell invasiveness **(E)**. ^*^ *p* < 0.05, ^**^ *p* < 0.01, ^***^ *p* < 0.001.

Furthermore, SLC16A1-AS1 was successfully silenced in MDA-MB-453 and BT474 cells with si-SLC16A1-AS1, which was confirmed by the qRT-PCR assay (**Figure 3A**). After SLC16A1-AS1 silencing, the viability (0–96 h), colony proliferation ability, and cell invasion and migration abilities were evaluated. The data indicated that SLC16A1-AS1 silencing significantly promoted cell viability time dependently (**Figure 3B**) as well as colony proliferation ability (**Figure 3C**) and cell migration (**Figure 3D**) and invasion (**Figure 3E**) abilities in both the MDA-MB-453 and BT474 cells.

### SLC16A1-AS1 Is a ceRNA of miR-552-5p in BC Cells

**Figure 4:**
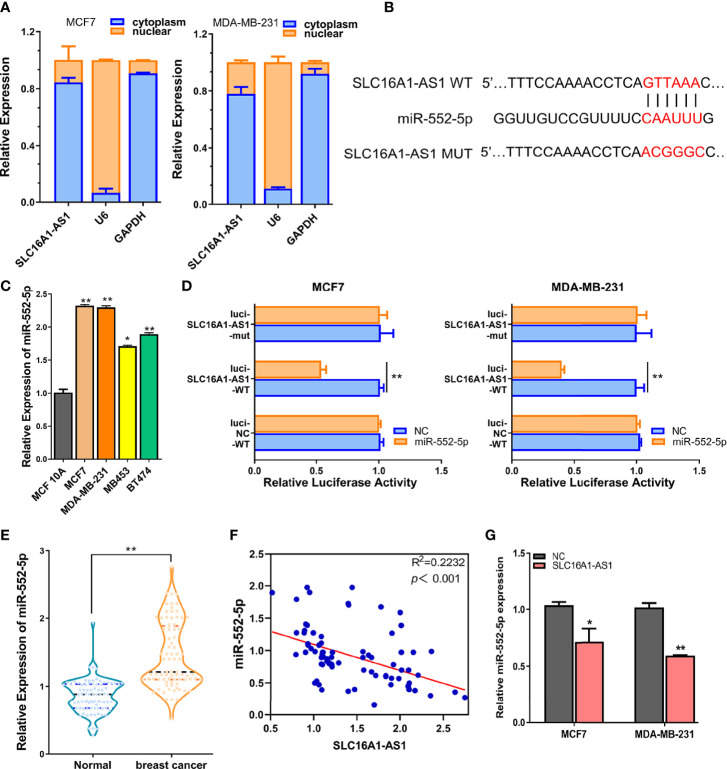
SLC16A1-AS1 is a sponge for miR-552-5p. **(A)** Cellular sublocalization of SLC16A1-AS1 in MCF7 and MDA-MB-231 cells was investigated with nuclear and cytoplasmic separation assay, and GAPDH and U6, respectively, acted as internal controls for cytoplasmic and nuclear expressions. **(B)** Diagram indicating the prospective binding sites for miR-552-5p with SLC16A1-AS1 predicted by miRDB (http://mirdb.org/) and mutated information of prospective miR-552-5p binding sites. **(C)** miR-552-5p expressions were significantly higher in MCF7 and MDA-MB-231 cells than in MCF 10A cells analyzed by qRT-PCR. **(D)** Dual-luciferase activities in MDA-MB-231 and MCF7 cells in the presence of negative control (NC), wild type (WT), or mutated (mut) reporter of SLC16A1-AS1 with miR-552-5p or without (NC) miR-552-5p mimics. **(E)** miR-552-5p expression was considerably higher in BC tissues versus the paired paracancerous tissues from 80 BC patients (the same specimens used in **Figure 1A**). **(F)** miR-552-5p expression level was significantly negatively related to SLC16A1-AS1 expression level in 80 BC tissues by Spearman correlation coefficient analysis (the same specimens used in **Figure 1A**). **(G)** miR-552-5p expression was confirmed to be negatively regulated by SLC16A1-AS1 expression after measuring miR-552-5p levels in MDA-MB-231 and MCF7 cells with SLC16A1-AS1 overexpression. ^*^ *p* < 0.05, ^**^ *p* < 0.01.

Increasing evidence has supported that cytoplasmic lncRNAs act as ceRNAs in regulating the biological functions and expressions of miRNAs (15, 16). Therefore, the cellular sublocalization expressions of SLC16A1-AS1 in MDA-MB-231 and MCF7 cells were investigated by extraction of the nuclear and cytoplasmic RNAs followed by qRT-PCR assay. The results demonstrated that SLC16A1-AS1 was distributed mostly in the cell cytoplasm than in the nuclear fraction (**Figure 4A**); consequently, we hypothesized that SLC16A1-AS1 could be a sponge to inhibit miRNAs by binding with their target mRNAs in BC development.

To verify this hypothesis, we first performed bioinformatics analysis to identify the possible miRNAs which might bind to SLC16A1-AS1 3′-UTR in BC cells. The 3′-UTR sequence of SLC16A1-AS1 was obtained by BLAST, and miR-552-5p was predicted to be potentially bound to SLC16A1-AS1 3′-UTR by searching miRDB (http://mirdb.org/). The diagram of prospective binding sites for miR-552-5p in SLC16A1-AS1 3′-UTR is shown in **Figure 4B**. We then investigated the functions of miR-552-5p in BC. miR-552-5p expression was found to be upregulated in BC cells (MCF7, MDA-MB-231, MB453, and BT474) versus the MCF 10A cells, and miR-552-5p expression was the highest in MDA-MB-231 and MCF7 cells (**Figure 4C**). Furthermore, dual-luciferase activity was conducted to validate the binding potential between SLC16A1-AS1 3′-UTR and miR-552-5p, after subcloning SLC16A1-AS1 3′-UTR with mutated or WT reporter gene into dual-luciferase reporter plasmid (pmirGLO), which indicated that the luciferase activities of the pmirGLO-SLC16A1-AS1 3′-UTR-WT reporter gene in both MCF7 and MDA-MB-231 cells were significantly decreased in the presence of miR-552-5p mimics (*p* < 0.01), while the luciferase activities were not affected after the predicted prospective miR-552-5p binding sites in SLC16A1-AS1 3′-UTR were mutated (**Figure 4D**).

Meanwhile, miR-552-5p expression in the cancerous tissues was significantly upregulated (**Figure 4E**, *p* < 0.01) and negatively correlated to SLC16A1-AS1 expression in the 80 BC patients included by Spearman correlation analysis (**Figure 4F**, *p* < 0.001), indicating that miR-552-5p acted as an oncogenic miRNA in BC.

Moreover, miR-552-5p expression was confirmed to be negatively regulated by SLC16A1-AS1 expression. As we can see in **Figure 4G**, miR-552-5p expressions in MDA-MB-231 and MCF7 cells were decreased with SLC16A1-AS1 overexpression.

Overall, these findings indicated that SLC16A1-AS1 was a ceRNA to regulate miR-552-5p in BC.

### SLC16A1-AS1 Sponges miR-552-5p to Upregulate WIF1 Expression in BC Cells

**Figure 5:**
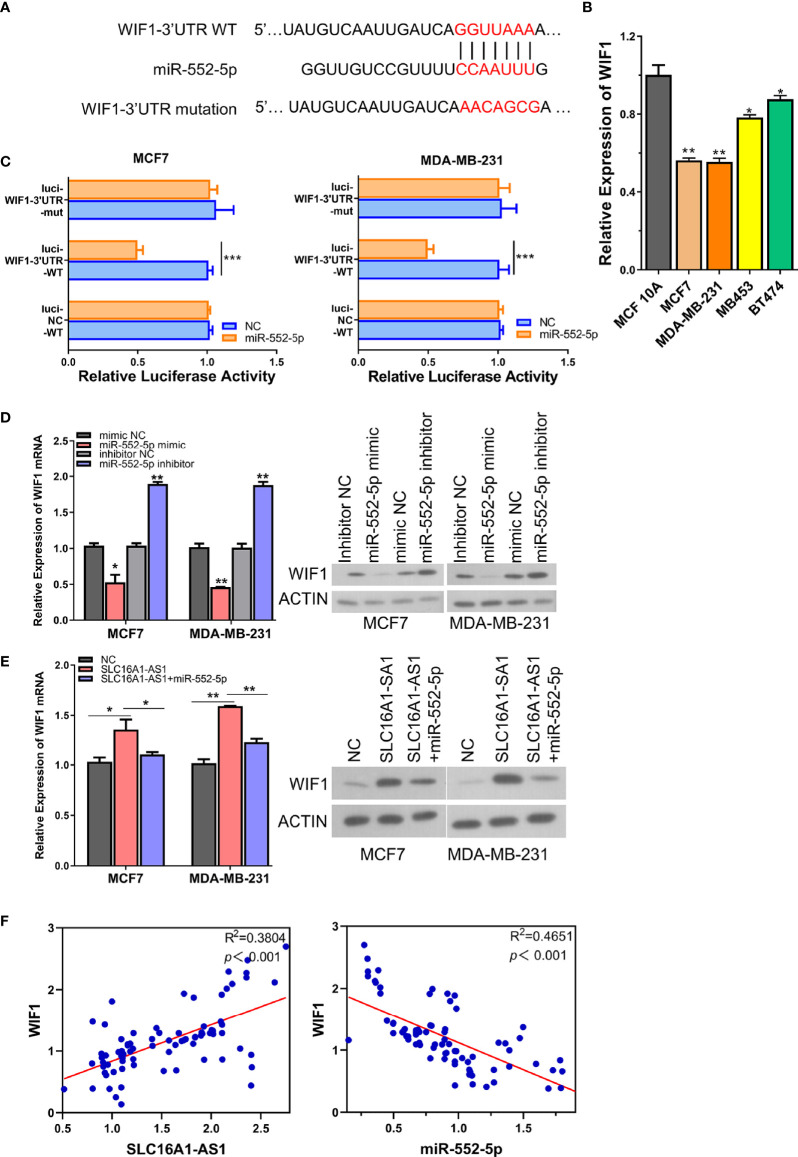
SLC16A1-AS1 increases WIF1 expression *via* sponging miR-552-5p. **(A)** Illustration of the prospective binding sites of miR-552-5p in WIF1 3′-UTR predicted with miRanda (http://www.microrna.org/). **(B)** mRNA expression of WIF1 was significantly lower in BC cells (MDA-MB-231 and MCF7) than in normal mammary gland epithelial cells (MCF 10A) analyzed by qRT-PCR. **(C)** Dual-luciferase activities in MDA-MB-231 and MCF7 cells with negative control (NC), wild type (WT), or mutated (mut) reporter of WIF1 3′-UTR with miR-552-5p or without (NC) miR-552-5p mimics. **(D)** mRNA and protein expressions of WIF1 in MCF7 and MDA-MB-231 cells after miR-552-5p was silenced (miR-552-5p inhibitor) or overexpressed (miR-552-5p mimics). **(E)** mRNA (left panel) and protein expressions (middle and right panels) of WIF1 in SLC16A1-AS1 overexpressed in MDA-MB-231 and MCF7 cells with or without co-transfecting miR-552-5p. **(F)** Spearman correlation assay of the correlation between WIF1 and SLC16A1-AS1 or miR-552-5p in BC tissues from 80 BC patients (the same specimens used in **Figure 1A**). ^*^ *p* < 0.05, ^**^ *p* < 0.01, ^***^ *p* < 0.001.

As an algorithm to identify the genomic targets of microRNAs, miRanda has been developed by the Computational Biology Center of Memorial Sloan-Kettering Cancer Center. After being analyzed with miRanda (http://www.microrna.org/), we found that WIF1 3′-UTR harbored the potential binding sites of miR-552-5p (**Figure 5A**). Studies have revealed that miR-552-5p promotes osteosarcoma cell development *via* targeting the WIF1 (17). However, whether WIF1 is a direct target of miR-552-5p in BC cells remains unknown. Therefore, we hypothesized that miR-552-5p may directly bind to WIF1 in BC cells.

To further confirm the direct binding between miR-552-5p and WIF1 3′-UTR, we compared the WIF1 expressions of MCF7, MDA-MB-231, MB453, and BT474 cells with MCF 10A cells, and the results revealed a significantly downregulated WIF1 expression in BC cells than in normal mammary gland epithelial cells (**Figure 5B**). The WT or mutated WIF1 3′-UTR reporter gene was then subcloned into pmirGLO. The luciferase activities of the WT WIF1 3′-UTR reporter gene in both MDA-MB-231 and MCF7 cells were significantly inhibited in the presence of the miR-552-5p mimics versus the miR-552-5p NC, while these activities did not change when the predicted binding sites of miR-552-5p in WIF1 3′-UTR were mutated (**Figures 5A, C**), representing that miR-552-5p directly targeted WIF1. This direct binding was verified by checking the protein and mRNA expressions of WIF1 in MDA-MB-231 and MCF7 cells in the presence of the miR-552-5p mimics or miR-552-5p inhibitor, which showed that the mRNA (**Figure 5D**, left panel) and protein (**Figure 5D**, middle and right panel) expressions of WIF1 were significantly inhibited by the miR-552-5p mimics but promoted by the miR-552-5p inhibitor.

Since the results above showed that SLC16A1-AS1 was a sponge of miR-552-5p, rescue experiments were performed to further find out whether SLC16A1-AS1 regulated WIF1 expressions in MDA-MB-231 and MCF7 cells by sponging miR-552-5p. The mRNA and protein expressions of WIF1 in SLC16A1-AS1 overexpressed in MDA-MB-231 and MCF7 cells with or without co-transfection of miR-552-5p mimics were first detected, which showed that SLC16A1-AS1 overexpression meaningfully upregulated WIF1 expressions in both the mRNA (**Figure 5E**, left panel) and protein (**Figure 5E**, middle and right panels) levels in MDA-MB-231 and MCF7 cells, while these changes were effectively rescued by co-transfecting miR-552-5p mimics.

Moreover, Spearman correlation analysis showed that WIF1 expression was positively correlated to SLC16A1-AS1 expression (**Figure 5F**) and negatively correlated to miR-552-5p expression (**Figure 5F**) in cancerous tissues from 80 BC patients. These findings indicated that WIF1 was upregulated after miR-552-5p was sponged by SLC16A1-AS1 in BC cells.

### The miR-552-5p Overexpression or WIF1 Silencing Reverses SLC16A1-AS1-Attenuated Aggressive Behaviors of BC

**Figure 6:**
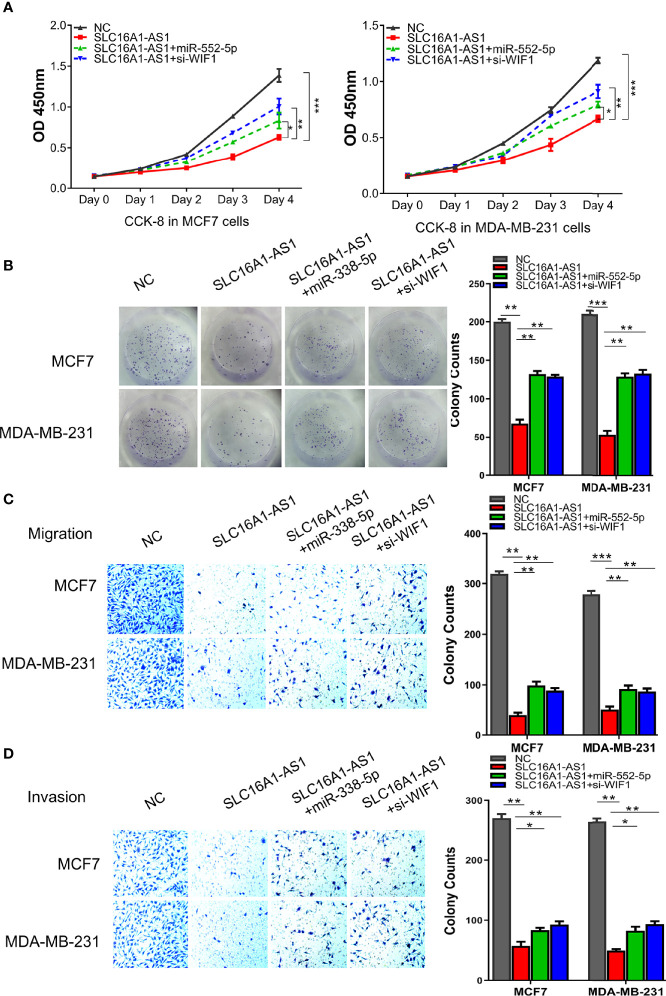
miR-552-5p overexpression or WIF1 knockdown reverses SLC16A1-AS1-attenuated BC aggressive behavior. To verify the functions of the SLC16A1-AS1/miR-552-5p/WIF1 axis in BC cells, the viability **(A)**, colony formation **(B)**, cell migration **(C)**, and cell invasion **(D)** in MDA-MB-231 and MCF7 cells after SLC16A1-AS1 overexpression (SLC16A1-AS1) with or without miR-552-5p overexpression by miR-552-5p mimics (miR-552-5p) or WIF1 silenced with small interfering RNA (si-WIF1) were studied, respectively. ^*^ *p* < 0.05, ^**^ *p* < 0.01, ^***^ *p* < 0.001.

Studies have reported that WIF1, as a secreted antagonist of Wnt proteins and an inhibitor of the Wnt signaling pathway (18–23), is a tumor suppressor in various malignancies (18); nevertheless, the mechanism of WIF1 in BC has not been fully clarified. Here, we performed the rescue experiments followed by analyzing the cell viability, the colony formation, and the migration and invasiveness abilities in MDA-MB-231 and MCF7 cells to further explore the functional roles of the SLC16A1-AS1/miR-552-5p/WIF1 axis in BC. The data indicated that SLC16A1-AS1 overexpression significantly suppressed OD values of MDA-MB-231 and MCF7 cells (0–4 days), and the inhibited OD values were partly reversed in the presence of the miR-552-5p mimics or si-WIF1 (**Figure 6A**). The colony formation abilities of MDA-MB-231 and MCF7 cells were also significantly decreased with SLC16A1-AS1 overexpression, which was partly rescued when the miR-552-5p mimics or si-WIF1 was co-transfected (**Figure 6B**). Transwell assays showed that cell migration (**Figure 6C**) and invasion (**Figure 6D**) abilities of MCF7 and MDA-MB-231 cells were all inhibited after SLC16A1-AS1 overexpression, which also significantly reversed when the miR-552-5p mimics or si-WIF1 was co-transfected. Therefore, the overexpressed miR-552-5p or the inhibited WIF1 reversed the preventative effect of SLC16A1-AS1 in BC development.

**Figure 7:**
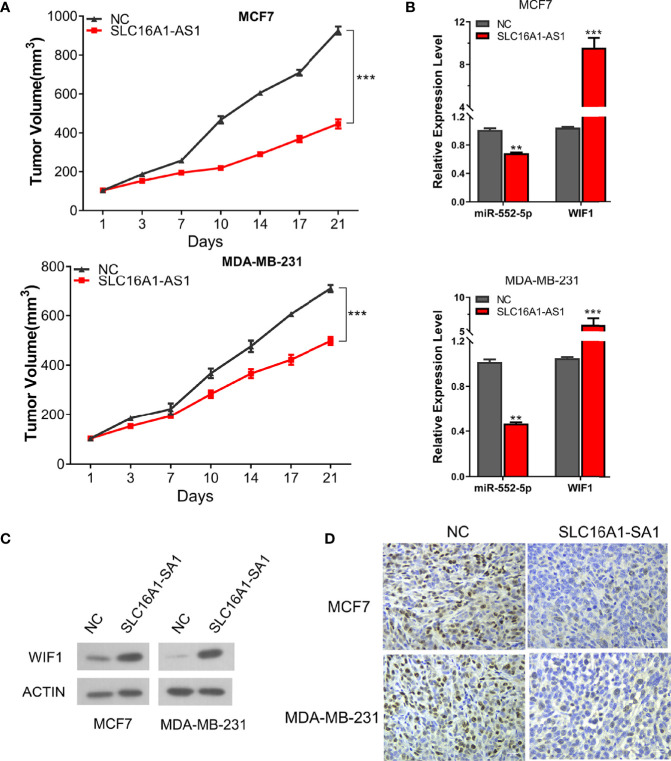
Overexpression of SLC16A1-AS1 in both MCF7 and MDA-MB-231 cells inhibits BC xenograft tumor growth by regulating the miR-552-5p/WIF1 axis in vivo. Xenograft tumor in nude mice was prepared with SLC16A1-AS1 overexpressed (SLC16A1-AS1) or the control (NC) plasmid-transfected MCF7 or MDA-MB-231 cells, then the following phenotypes were analyzed: **(A)** growth curve (tumor volume). **(B)** miR-552-5p and WIF1 mRNA expressions via the qRT-PCR assay. ^*^ *p* < 0.05, ^**^ *p* < 0.01, ^***^ *p* < 0.001. **(C)** WIF1 protein expression via the Western blot assay. **(D)** Ki-67 protein expression by the immunochemistry assay.

Furthermore, xenograft models generated by the subcutaneous injection of SLC16A1-AS1 stably overexpressed MDA-MB-231 or MCF7 cells were then applied to investigate the biological function of the SLC16A1-AS1/miR-552-5p/WIF1 axis *in vivo*. Similar to the *in-vitro* results, SLC16A1-AS1 overexpression considerably inhibited tumor volume than in the NC group (**Figure 7A**). Moreover, SLC16A1-AS1 overexpression significantly inhibited miR-552-5p expression (**Figure 7B**), while it increased WIF1 expression (**Figure 7C**) in the xenograft tissues. Meanwhile, the immunohistochemistry assay confirmed that SLC16A1-AS1 overexpression decreased Ki-67 expression (**Figure 7D**), indicating a reduced cell proliferation.

Taken together, our findings revealed that SLC16A1-AS1 was a tumor suppressor and inhibited the tumorigenesis and highly aggressive behaviors of BC cells *via* sponging miR-552-5p to activate WIF1 both *in vitro* and *in vivo*.

## Discussion

Despite great developments in the clinical management of BC, it still ranks the fifth leading cause of global cancer mortality in 2020. This makes it urgent to systematically understand the molecular mechanisms involved in BC carcinogenesis and metastasis, thus recognizing novel targets to improve the clinical outcomes of BC.

With the development of high-throughput sequencing technologies, over 80% of genes are identified to be non-coding RNAs in the human genome, such as lncRNAs and miRNAs, which were initially thought to be transcriptional “noise” (24, 25). Accumulated studies have reported that lncRNAs are critical in BC progression, and have focused on discovering novel diagnostic, therapeutic, and prognostic targets for BC from lncRNAs (26–28).

By reviewing the literature, LC16A1-AS1 has been reported to enhance radiosensitivity and repress cell proliferation and invasion by regulating the miR-301b-3p/CHD5 axis in hepatocellular carcinoma (11) and to suppress cell proliferation in cervical squamous cell carcinoma by the miR194/SOCS2 axis (12). Our qRT-PCR assay showed that SLC16A1-AS1 overexpression significantly downregulated miR194 expression but upregulated SOCS2 expression, indicating that SLC16A1-AS1 may also suppress BC by the miR194/SOCS2 axis (**Supplementary Figure 1**).

In the present study, we first identified that SLC16A1-AS1 was downregulated significantly in BC cells and tissues. This finding was in line with the data from the TCGA datasets, indicating that SLC16A1-AS1 was a potential tumor suppressor in BC. Moreover, the Kaplan–Meier method revealed a poor prognosis with lower SLC16A1-AS1 expression in BC patients, which was considerably related to decreased overall survival rate and disease-free survival rate, indicating that it was a possible prognostic factor and a therapeutic target for BC patients.

The biological roles of lncRNAs are fundamentally dependent on their distribution in the cytoplasm and/or nucleus. Increasing evidence has shown that lncRNAs being expressed in the cytoplasmic fraction are involved in the post-transcriptional gene regulation and may function as ceRNAs to protect the target mRNAs from degradation (2, 29, 30). By extracting cell cytoplasmic/nuclear fractions followed by qRT-PCR assay, we identified that SLC16A1-AS1 was predominantly expressed in the cytoplasm, suggesting the possibility for it to act as an miRNA sponge. Consequently, we further investigated miRNA sponged by SLC16A1-AS1 and miRNA targeting mRNA. Bioinformatics exploration displayed that miR-552-5p was potentially sponged by SLC16A1-AS1 and WIF1 was the target gene of miR-552-5p, which were confirmed by dual-luciferase activity assay, Spearman correlation analysis, gain and loss of function manipulation, and rescue experiments.

As a result, our current work offered novel evidence supporting that SLC16A1-AS1 acted as a ceRNA of miR-552-5p to upregulate WIF1 expression. Since the function of the Wnt signaling pathway is important in BC carcinogenesis and development (31, 32), and WIF1 is an antagonist of the Wnt signaling pathway (33), our findings revealed a therapeutic implication of the SLC16A1-AS1/miR-552-5p/WIF1 pathway in BC patients.

## Conclusion

This work disclosed that SLC16A1-AS1 was a tumor suppressor in BC, and lower SLC16A1-AS1 expression was related to poor prognosis of BC patients. In terms of functions and mechanisms, SLC16A1-AS1 inhibited the viability, colony formation, invasion, and migration of BC cells *via* sponging miR-552-5p to upregulate WIF1 expression. Our findings provide a better understanding of the biological functions and mechanisms of SLC16A1-AS1 to serve as a new tumor suppressor, a ceRNA of miR-552-5p, and a prognostic predictor of BC. Therefore, targeting the SLC16A1-AS1/miR-552-5p/WIF1 axis would offer novel strategies for the diagnosis, therapy, and prognosis of BC patients.

## Data Availability Statement

The original contributions presented in the study are included in the article/**Supplementary Material**. Further inquiries can be directed to the corresponding author.

## Ethics Statement

Written informed consents were obtained from all individuals who participated in this study.

## Author Contributions

XZ designed the experiments. BJ conducted the experiments. JX analyzed the data. BJ wrote the manuscript. BJ and XZ revised the manuscript. All authors read and approved the final manuscript.

## Conflict of Interest

The authors declare that the research was conducted in the absence of any commercial or financial relationships that could be construed as a potential conflict of interest.

## Publisher’s Note

All claims expressed in this article are solely those of the authors and do not necessarily represent those of their affiliated organizations, or those of the publisher, the editors and the reviewers. Any product that may be evaluated in this article, or claim that may be made by its manufacturer, is not guaranteed or endorsed by the publisher.

## Supplementary Material

Supplementary Figure 1 Expressions of miR194 and SOCS2 after SLC16A1-AS1 overexpression in BC cells by qRT-PCR assay.

## References

[1] Sung H Ferlay J Siegel RL Laversanne M Soerjomataram I Jemal A. Global Cancer Statistics 2020: GLOBOCAN Estimates of Incidence and Mortality Worldwide for 36 Cancers in 185 Countries. CA: Cancer J Clin (2021) 71(3):209–49. doi: 10.3322/caac.21660 33538338

[2] Liang Y Song X Li Y Chen B Zhao W Wang L. LncRNA BCRT1 Promotes Breast Cancer Progression by Targeting miR-1303/PTBP3 Axis. Mol Cancer (2020) 19:85. doi: 10.1186/s12943-020-01206-5 32384893 PMC7206728

[3] Djebali S Davis CA Merkel A Dobin A Lassmann T Mortazavi A. Landscape of Transcription in Human Cells. Nature (2012) 489:101–8. doi: 10.1038/nature11233 PMC3684276 22955620

[4] Chang KC Diermeier SD Yu AT Brine LD Russo S Bhatia S. MaTAR25 lncRNA Regulates the Tensin1 Gene to Impact Breast Cancer Progression. Nat Commun (2020) 11:6438. doi: 10.1038/s41467-020-20207-y 33353933 PMC7755919

[5] Gupta RA Shah N Wang KC Kim J Horlings HM Wong DJ. Long non-Coding RNA HOTAIR Reprograms Chromatin State to Promote Cancer Metastasis. Nature (2010) 464:1071–6. doi: 10.1038/nature08975 PMC3049919 20393566

[6] Salmena L Poliseno L Tay Y Kats L Pandolfi PP. A ceRNA Hypothesis: The Rosetta Stone of a Hidden RNA Language? Cell (2011) 146:353–8. doi: 10.1016/j.cell.2011.07.014 PMC3235919 21802130

[7] Tay Y Rinn J Pandolfi PP. The Multilayered Complexity of ceRNA Crosstalk and Competition. Nature (2014) 505:344–52. doi: 10.1038/nature12986 PMC4113481 24429633

[8] Liu HY Lu SR Guo ZH Zhang ZS Ye X Du Q. lncRNA SLC16A1-AS1 as a Novel Prognostic Biomarker in non-Small Cell Lung Cancer. J Investig Med (2020) 68:52–9. doi: 10.1136/jim-2019-001080 PMC6996107 31371390

[9] Feng H Zhang X Lai W Wang J. Long non-Coding RNA SLC16A1-AS1: Its Multiple Tumorigenesis Features and Regulatory Role in Cell Cycle in Oral Squamous Cell Carcinoma. Cell Cycle (2020) 19:1641–53. doi: 10.1080/15384101.2020.1762048 PMC7469610 32450050

[10] Logotheti S Marquardt S Gupta SK Richter C Edelhauser BAH Engelmann D. LncRNA-SLC16A1-AS1 Induces Metabolic Reprogramming During Bladder Cancer Progression as Target and Co-Activator of E2F1. Theranostics (2020) 10:9620–43. doi: 10.7150/thno.44176 PMC7449907 32863950

[11] Pei S Chen Z Tan H Fan L Zhang B Zhao C. SLC16A1-AS1 Enhances Radiosensitivity and Represses Cell Proliferation and Invasion by Regulating the miR-301b-3p/CHD5 Axis in Hepatocellular Carcinoma. Environ Sci Pollut Res Int (2020) 27:42778–90. doi: 10.1007/s11356-020-09998-1 32748357

[12] Zhang H Jin S Ji A Ma Y Zhang C Wang A. LncRNA SLC16A1-AS1 Suppresses Cell Proliferation in Cervical Squamous Cell Carcinoma (CSCC) Through the miR-194/SOCS2 Axis. Cancer Manag Res (2021) 13:1299–306. doi: 10.2147/CMAR.S276629 PMC7884948 33603475

[13] Wu D Zhu J Fu Y Li C Wu B. LncRNA HOTAIR Promotes Breast Cancer Progression Through Regulating the miR-129-5p/FZD7 Axis. Cancer biomark (2021) 2:203–12. doi: 10.3233/CBM-190913 PMC12499983 33104019

[14] Li F Fountzilas C Puzanov I Attwood KM Morrison C Ling X. MEG3 Overexpression Inhibits the Tumorigenesis of Breast Cancer by Downregulating miR-21 Through the PI3K/Akt Pathway. Arch Biochem Biophys (2019) 661:22–30. doi: 10.1016/j.abb.2018.10.021 30389444

[15] Cao C Zhang T Zhang D Xie L Zou X Lei L. The Long non-Coding RNA, SNHG6-003, Functions as a Competing Endogenous RNA to Promote the Progression of Hepatocellular Carcinoma. Oncogene (2017) 36:1112–22. doi: 10.1038/onc.2016.278 27530352

[16] Qu L Ding J Chen C Wu ZJ Liu B Gao Y. Exosome-Transmitted lncARSR Promotes Sunitinib Resistance in Renal Cancer by Acting as a Competing Endogenous RNA. Cancer Cell (2016) 29:653–68. doi: 10.1016/j.ccell.2016.03.004 27117758

[17] Cai W Xu Y Yin J Zuo W Su Z. miR-552-5p Facilitates Osteosarcoma Cell Proliferation and Metastasis by Targeting WIF1. Exp Ther Med (2019) 17:3781–8. doi: 10.3892/etm.2019.7361 PMC6447863 30988764

[18] Kerekes K Banyai L Trexler M Patthy L. Structure, Function and Disease Relevance of Wnt Inhibitory Factor 1, a Secreted Protein Controlling the Wnt and Hedgehog Pathways. Growth Factors (2019) 37:29–52. doi: 10.1080/08977194.2019.1626380 31210071

[19] Poggi L Casarosa S Matthias C. An Eye on the Wnt Inhibitory Factor Wif1. Front Cell Dev Biol (2018) 6:167. doi: 10.3389/fcell.2018.00167 30574494 PMC6292148

[20] Wei Y Ma H Zhou H Yin H Yang J Song Y. miR-424-5p Shuttled by Bone Marrow Stem Cells-Derived Exosomes Attenuates Osteogenesis *via* Regulating WIF1-Mediated Wnt/beta-Catenin Axis. Aging (2021) 13:17190–201. doi: 10.18632/aging.203169 PMC8312462 34229300

[21] Yao X Mao Y Wu D Zhu Y Lu J Huang Y. Exosomal Circ_0030167 Derived From BM-MSCs Inhibits the Invasion, Migration, Proliferation and Stemness of Pancreatic Cancer Cells by Sponging miR-338-5p and Targeting the Wif1/Wnt8/beta-Catenin Axis. Cancer Lett (2021) 512:38–50. doi: 10.1016/j.canlet.2021.04.030 33971282

[22] Lu C Shao X Zhou S Pan C. LINC00176 Facilitates CD4(+)T Cell Adhesion in Systemic Lupus Erythematosus *via* the WNT5a Signaling Pathway by Regulating WIF1. Mol Immunol (2021) 134:202–9. doi: 10.1016/j.molimm.2021.02.018 33813201

[23] Pennarubia F Pinault E Al Jaam B Brun CE Maftah A Germot A. Mouse WIF1 Is Only Modified With O-Fucose in Its EGF-Like Domain III Despite Two Evolutionarily Conserved Consensus Sites. Biomolecules (2020) 10:1250. doi: 10.3390/biom10091250 PMC7565927 32872229

[24] Schmitt AM Chang HY. Long Noncoding RNAs in Cancer Pathways. Cancer Cell (2016) 29:452–63. doi: 10.1016/j.ccell.2016.03.010 PMC4831138 27070700

[25] Louro R Smirnova AS Verjovski-Almeida S. Long Intronic Noncoding RNA Transcription: Expression Noise or Expression Choice? Genomics (2009) 93:291–8. doi: 10.1016/j.ygeno.2008.11.009 19071207

[26] Zhou D Ren K Wang M Wang J Li E Hou C. Long non-Coding RNA RACGAP1P Promotes Breast Cancer Invasion and Metastasis *via* miR-345-5p/RACGAP1-Mediated Mitochondrial Fission. Mol Oncol (2021) 15:543–59. doi: 10.1002/1878-0261.12866 PMC7858103 33252198

[27] Liang ZZ Zhu RM Li YL Jiang HM Li RB Wang Q. Differential Epigenetic Profiles Induced by Sodium Selenite in Breast Cancer Cells. J Trace Elem Med Biol (2021) 64:126677. doi: 10.1016/j.jtemb.2020.126677 33246299

[28] Zhou T Lin K Nie J Pan B He B Pan Y. LncRNA SPINT1-AS1 Promotes Breast Cancer Proliferation and Metastasis by Sponging Let-7 a/B/I-5p. Pathol Res Pract (2021) 217:153268. doi: 10.1016/j.prp.2020.153268 33246290

[29] Cesana M Cacchiarelli D Legnini I Santini T Sthandier O Chinappi M. A Long Noncoding RNA Controls Muscle Differentiation by Functioning as a Competing Endogenous RNA. Cell (2011) 147:358–69. doi: 10.1016/j.cell.2011.09.028 PMC3234495 22000014

[30] Lalevee S Feil R. Long Noncoding RNAs in Human Disease: Emerging Mechanisms and Therapeutic Strategies. Epigenomics (2015) 7:877–9. doi: 10.2217/epi.15.55 26418705

[31] Kar S Jasuja H Katti DR Katti KS. Wnt/beta-Catenin Signaling Pathway Regulates Osteogenesis for Breast Cancer Bone Metastasis: Experiments in an In Vitro Nanoclay Scaffold Cancer Testbed. ACS Biomater Sci Eng (2020) 6:2600–11. doi: 10.1021/acsbiomaterials.9b00923 33463270

[32] Nie J Jiang HC Zhou YC Jiang B He WJ Wang YF. MiR-125b Regulates the Proliferation and Metastasis of Triple Negative Breast Cancer Cells *via* the Wnt/beta-Catenin Pathway and EMT. Biosci Biotechnol Biochem (2019) 83:1062–71. doi: 10.1080/09168451.2019.1584521 30950326

[33] Poggi L Casarosa S Carl M. An Eye on the Wnt Inhibitory Factor Wif1. Front Cell Dev Biol (2018) 6:167. doi: 10.3389/fcell.2018.00167 30574494 PMC6292148

