# Supplementary figures and images for: Overexpression of lncRNA SLC16A1-AS1 Suppresses the Growth and Metastasis of Breast Cancer via the miR-552-5p/WIF1 Signaling Pathway
Source: Front Oncol. 2022 Mar 15;12:712475. doi: 10.3389/fonc.2022.712475 (PMC8964943; doi:10.3389/fonc.2022.712475)

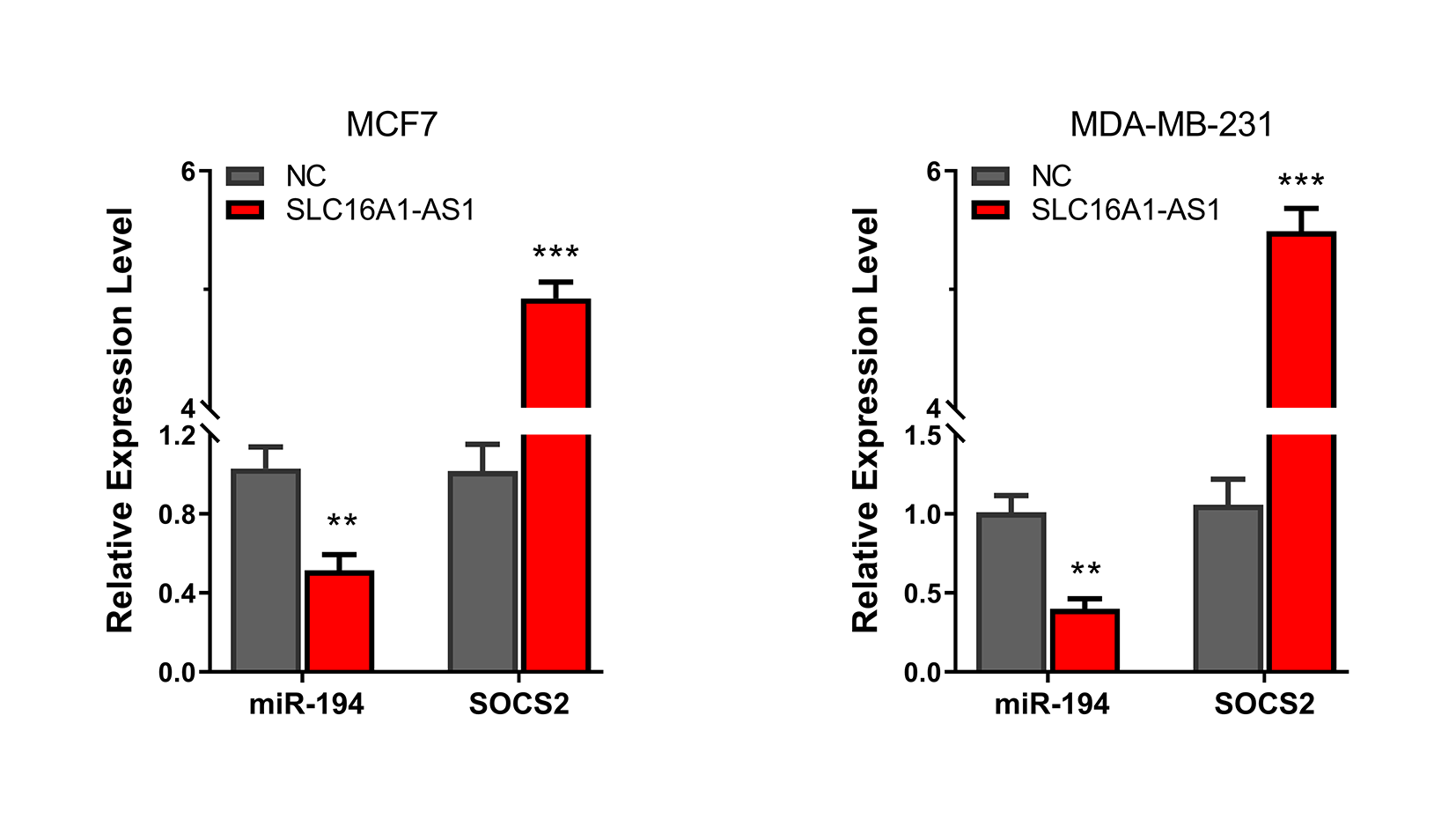

Supplement: Supplementary Figure 1 — Expressions of miR194 and SOCS2 after SLC16A1-AS1 overexpression in BC cells by qRT-PCR assay. [file Image_1.tif]
