# Supplementary material for: Overexpression of lncRNA SLC16A1-AS1 Suppresses the Growth and Metastasis of Breast Cancer via the miR-552-5p/WIF1 Signaling Pathway
Source: Front Oncol. 2022 Mar 15;12:712475. doi: 10.3389/fonc.2022.712475 (PMC8964943; doi:10.3389/fonc.2022.712475)
Supplement: Supplementary file 2 [file Table_1.docx]

Supplementary Table 1. The realtime PCR primers for the target genes

| Gene name | Realtime PCR primer |
| --- | --- |
| miR-552-5p | F 5′-GTTTAACCTTTTGCCTGTTGG-3′  R 5′-CGAACGCTTCACGAATTTG-3′ |
| SLC16A1-AS1 | F 5′- GGGAGACTTAGGCACAAATTAACC  R 5′- ATGTTGGTGTGCTTGAAATCTTCC |
| WIF1 | F 5′-TTGTTTCAGTGCTTTGGGACAG-3′  R 5′-CCCCCAGACACCATAAATGC-3′ |
| U6 | F 5′-CTCGCTTCGGCAGCACATATAC  R 5′-AACGCTTCACGAATTTGCGTGTC |
| GAPDH | F 5′-TCATGGGTGTGAACCATGAGAA-3′  R 5′-GGCATGGACTGTGGTCATGAG-3′ |
