# Supplementary material for: Overexpression of lncRNA SLC16A1-AS1 Suppresses the Growth and Metastasis of Breast Cancer via the miR-552-5p/WIF1 Signaling Pathway
Source: Front Oncol. 2022 Mar 15;12:712475. doi: 10.3389/fonc.2022.712475 (PMC8964943; doi:10.3389/fonc.2022.712475)
Supplement: Supplementary file 3 [file Table_2.docx]

Supplementary table 2. Correlations of SLC16A1-AS1 expression with clinicopathologic features of breast cancer

| Variable | Number | SLC16A1-AS1 expression | | *P*-value |
| --- | --- | --- | --- | --- |
|  |  | Low | High |  |
| Age (years) |  |  |  | 0.564 |
| < 60 | 29 | 15 | 14 |  |
| ≥ 60 | 51 | 25 | 26 |  |
| Gender |  |  |  | 0.93 |
| Male | 10 | 4 | 6 |  |
| Female | 70 | 36 | 34 |  |
| Tumor size (cm) |  |  |  | 0.03 |
| < 3 | 19 | 6 | 23 |  |
| ≥ 3 | 61 | 34 | 17 |  |
| TNM |  |  |  | 0.02 |
| I-II | 39 | 11 | 29 |  |
| III-IV | 41 | 29 | 11 |  |
| Lymph nodes metastasis |  |  |  | 0.005 |
| No | 42 | 12 | 30 |  |
| Yes | 38 | 28 | 10 |  |
| Differentiation |  |  |  | 0.437 |
| High | 32 | 22 | 10 |  |
| Low | 48 | 23 | 25 |  |
| ER status |  |  |  | 0.432 |
| Positive | 35 | 19 | 16 |  |
| Negative | 45 | 23 | 22 |  |
| PR status |  |  |  | 0.324 |
| Positive | 40 | 17 | 23 |  |
| Negative | 30 | 9 | 21 |  |
| HER-2 status |  |  |  | 0.689 |
| Positive | 22 | 7 | 15 |  |
| Negative | 48 | 17 | 31 |  |
| WIF1 expression |  |  |  | 0.004 |
| low | 45 | 7 | 38 |  |
| high | 35 | 33 | 2 |  |
